# Wellness Interventions in Emergency Medicine Residency Programs: Review of the Literature Since 2017

**DOI:** 10.5811/westjem.2020.11.48884

**Published:** 2020-12-19

**Authors:** Arlene Chung, Sarah Mott, Katie Rebillot, Simiao Li-Sauerwine, Sneha Shah, Wendy C. Coates, Lalena M. Yarris

**Affiliations:** * Maimonides Medical Center, Department of Emergency Medicine, Brooklyn, New York; † Emergency Care Consultants, Minneapolis, Minnesota; ‡ LAC + USC Medical Center, Department of Emergency Medicine, Los Angeles, California; § The Ohio State University, Department of Emergency Medicine, Columbus, Ohio; ¶ UCLA David Geffen School of Medicine, Department of Emergency Medicine, Los Angeles, California; || Oregon Health & Science University, Department of Emergency Medicine, Portland, Oregon

## Abstract

**Introduction:**

Recent research demonstrates burnout prevalence rates as high as 76% in emergency medicine (EM) residents. In 2017 the Accreditation Council for Graduate Medical Education (ACGME) required that all training programs provide dedicated wellness education for their trainees as a requirement for accreditation. We aimed to conduct a systematic review of published wellness interventions conducted in EM residency programs following the implementation of the 2017 ACGME Common Program Requirements change in order to characterized published intervention and evaluate their effectiveness.

**Methods:**

We applied a published approach to conducting systematic reviews of the medical education literature. We performed a search of the literature from January 1, 2017–February 1, 2020. Studies were included for final review if they described a specific intervention and reported outcomes with the primary goal of improving EM resident wellness. Outcomes were characterized using the Kirkpatrick training evaluation model.

**Results:**

Eight of 35 identified studies met inclusion criteria. Most described small convenience samples of EM residents from single training programs and used the satisfaction rates of participants as primary outcome data. Only quantitative assessment methods were used. Studies addressed only a limited number of factors affecting resident wellness. The majority of interventions focused on personal factors, although a few also included sociocultural factors and the learning and practice environment.

**Conclusion:**

There is a relative dearth of literature in the area of research focused on interventions designed to improve EM resident wellness. Furthermore, the studies we identified are narrow in scope, involve relatively few participants, and describe programmatic changes of limited variety. Future directions include an increase and emphasis on multi-institutional studies, randomized controlled trials, qualitative methodology, and opportunities for funded research.

## INTRODUCTION

Emergency medicine (EM) is a high-stakes, fast-paced specialty requiring quick decision-making with limited information and often with critically ill patients. High anxiety and concern for poor outcomes influences emotional exhaustion and burnout among emergency physicians.^1^ Other factors that uniquely contribute to burnout in emergency physicians include unpredictable and inconsistent shift schedules^2^; sleep cycle disturbances^2^; increasing administrative burden without corresponding autonomy^3^; and expanding requirements around documentation within the electronic health record (EHR).^4^

Residency is one of the most physically and mentally taxing periods of a physician’s career. Compared with attending physicians or medical students, residents score significantly lower on measures of self-care including sleep, exercise, seatbelt use, and overall wellness.^5^ Factors related to burnout begin to take effect during intern year of residency secondary to issues such as duty hours, lack of autonomy, sleep deprivation, and resident mistreatment.^6^–^8^ These stressors are further compounded by the need to rapidly develop clinical competence in a setting with complex and sometimes critically ill patients, and low pay relative to hours worked.

In addition, residents have cited that these factors negatively impact their interactions and relationships with patients and have increased frequency of conflict with colleagues.^9^ This potential erosion of personal wellness during training can profoundly impact patient care. Recent studies suggest that EM attending physicians experience burnout rates greater than 70% as defined by the Maslach Burnout Inventory (MBI).^1^ EPs in training have been demonstrated to be equally susceptible. In a 2014 study involving residents from eight EM programs, 65% met criteria for burnout.^3^ A more recent large-scale prevalence study of over 1500 EM residents demonstrated the prevalence of burnout to be even higher, at 76.1%.^10^

In 2017 the Accreditation Council for Graduate Medical Education (ACGME) implemented common core program requirements focusing on the psychological, emotional, and physical well-being of residents.^11^ Current and recently published research has focused primarily on factors contributing to burnout^12^; less is known about the efficacy of interventions to mitigate burnout and improve wellness among EM trainees. Moreover, to date there are no widely accepted uniform recommendations regarding best practices for wellness interventions within residency programs.

We conducted a systematic review of published studies that describe interventions to mitigate burnout or improve wellness in EM training programs in order to characterize the published interventions and evaluate their effectiveness as measured by outcomes. Understanding the current state of the literature and remaining gaps is crucial as educators seek to design and implement programs intended to meaningfully combat burnout and improve wellness among trainees. In this systematic review, we focused our investigation on wellness interventions conducted in EM residency programs following the implementation of the 2017 ACGME Common Program Requirements.

## METHODS

Using the methodology described by Cook and West,^13^ we conducted a systematic review of the literature published within the MEDLINE database for articles published between January 1, 2017–February 1, 2020. This starting date was chosen to coincide with the implementation of the updated 2017 ACGME Common Program requirements that explicitly require programs’ support of resident well-being.^14^ We used the following search terms: wellness, well-being, burnout, and resilience. Search parameters were limited to EM residents and EM residency programs. Articles describing the results of primary studies of wellness interventions were included for final analysis. We excluded abstracts, opinion pieces, and literature reviews. Bibliographies of relevant articles were reviewed to identify any additional studies.

Articles identified through these search methods were screened by two authors and included for final review if they described a specific intervention and reported a primary outcome that addressed the need for supporting resident well-being in accordance with the ACGME Common Program Requirements Section VI.C.^14^ For those that met these criteria, we collected additional details including study design, population, type of intervention, a brief description of the intervention, outcome measure(s), and primary findings. Outcomes were characterized using the Kirkpatrick training evaluation model, which describes outcomes using increasing levels of complexity and potential impact: Level 1 (reaction); Level 2 (learning); Level 3 (behavior); and Level 4 (outcomes). We considered a change in burnout, perceived wellness, or other related outcome as assessed by a tool with existing validity evidence to be a Level 4.

## RESULTS

We identified 35 articles on our initial search of the literature. No additional studies meeting the inclusion criteria described above were found after a review of references cited. Eight published studies were included for final review based on our inclusion criteria (Table 1).

An additional six articles described specific wellness interventions but did not provide outcome data, and although these were not considered in our analysis, we have provided them for reference (Appendix 1).

### Study Participants

The majority of studies (6 of 8) took place in a single residency program using a convenience sample ranging from 24–58 with an average of 34 residents.^15^–^20^ One of the multi-institutional studies included two EM programs (n = 20) and the other included 10 EM programs (n = 437).^21^,^22^ All of the studies included EM residents at all postgraduate training levels. None of the studies conducted a power analysis to determine the number of participants required to demonstrate a significant effect of the intervention.

### Types of Interventions

The most commonly encountered wellness intervention (5 of 8) was a formal, classroom-based initiative.^15^,^16^,^18^,^20^,^22^ Three of these studies described a year-long curriculum^16^,^18^,^22^ and two described a single session.^15^,^20^ All five provided a broad overview of wellness during residency training and attempted to address multiple facets including work-life balance, stress management, and burnout coping mechanisms. The remaining three interventions included a peer support program,^19^ a virtual storytelling platform,^17^ and wearable fitness monitors to track sleep, steps, and activity.^21^

### Measured Outcomes

Included studies used a variety of previously published measures with existing validity evidence to assess the impact of wellness interventions (Table 2). Two studies used the MBI-HSS^18^,^22^; one used the Perceived Wellness Scale 21; and one used a combination of the Brief Resident Wellness Profile and Short Form Health Survey (SF-8).^16^ Many of the studies also used satisfaction surveys to assess participant reactions to the interventions,^15^–^20^ and two used a combination of both.^16^,^18^

### Main Findings

A diverse array of interventions were described in our literature review. Overall, participant satisfaction rates for changes aimed at improving resident wellness were high across all of the studies included for analysis, regardless of the specific intervention employed. Although the specific assessment tools varied, none of the authors demonstrated statistically significant differences between rates of wellness and/or burnout before and after the intervention period. Lastly, none of the studies included in our final analysis received grant support or other funding.

## DISCUSSION

While EM as a specialty has responded with large-scale wellness initiatives, such as the American College of Emergency Physicians’ Wellness Week and the formation of dedicated wellness committees within major national organizations, there is still a paucity of understanding about interventions employed for individual programs and learners. In our review of the literature since 2017, when the ACGME mandated that programs include wellness-specific programming, we found eight published studies that describe implementation of interventions designed to improve resident wellness in EM residency programs. The majority of studies describe small convenience samples of EM residents from single training programs and used the satisfaction rates of participants as primary outcome data. While the majority of outcomes are modest in size and limited in impact, these studies demonstrate encouraging pilot data to inform larger scale studies.

Expert consensus supports the development of a more comprehensive model in order to effect positive change. One such model proposed by the National Academy of Medicine Model of Clinician Well-Being describes seven important factors: society and culture; rules and regulations; organizational factors; learning and practice environment; healthcare responsibilities; personal factors; and skills and abilities.^12^ Only the last two factors are considered intrinsic to the individual. Although the majority of interventions studied focus on individual factors, a few also included sociocultural factors and the learning and practice environment. Aside from the limited number of factors mentioned, none of the interventions addressed systems-based challenges, such as the EHR, clerical burden, initial licensure and maintenance of certification, litigation risk, or regulatory requirements. These are areas that are ripe for additional investigation.

Our review of the literature suggests a need for a more diverse field of interventions that aim to mitigate the risk of burnout and promote resident engagement. The current literature supports a comprehensive and longitudinal focus on physician well-being that begins in residency training and emphasizes environmental factors, more specifically the following: that programs create and employ interventions to promote well-being; enact systems to better recognize depression, stress and burnout among residents; and fortify trainees in building resilience. Supportive experiences and healthy coping skills learned during training produce resilient residents and pave the way for career longevity and professional satisfaction.

High-quality published research studies of measurable outcomes with large samples of learners are needed to inform educators about which wellness interventions improve trainee outcomes. These studies are time and labor intensive, and would be supported and facilitated by dedicated grant funding for wellness research from national organizations and local entities. Our systematic review of wellness initiatives suggests that the next steps toward understanding how to meaningfully impact trainee wellness are to support multi-institutional studies, randomized controlled trials, and qualitative studies to further explore the phenomenon of wellness in medical education, and opportunities for funded research.

## LIMITATIONS

The search window of articles published since January 1, 2017, is narrow and we may not have captured relevant wellness interventions published prior to that date. However, this date is significant as it marks the year when the ACGME Common Program Requirements began to mandate wellness initiatives for residency program accreditation. The goal of this literature review was to identify the highest quality published evidence and is not meant to be a comprehensive review of all interventions. We did not include a review of abstracts or the gray literature, such as conference proceedings, reports, or dissertations, as we wished to capture the best-studied and most effective interventions as reflected in peer-reviewed manuscript publication.

The methodology described within this small pool of literature is limited; thus, we were unable to draw definitive conclusions regarding the overall effectiveness and generalizability of interventions. Although the Kirkpatrick model is widely accepted as a method of categorizing educational outcomes, the level of outcome that matters may vary depending on the study aim. For educational concepts where learner reactions and perceptions are the outcome of interest, Level 1 outcomes may be the “best” outcomes to measure; thus, Kirkpatrick levels are not a definitive hierarchy of quality. The majority of studies described small convenience samples of EM residents from single training programs and used the satisfaction rates of participants as primary outcome data. Additionally, none of the studies we identified primarily used qualitative methods of assessment, which offer a richer understanding of resident experiences and can serve to guide the focus of quantitative studies.

## CONCLUSION

Despite the increased attention to EM resident wellness, there currently exists only a modest number of published studies describing wellness interventions for EM residents with variable outcome data primarily focused on participant satisfaction. This review of the literature reflects a need for scholarship of EM resident wellness to extend beyond the current focus on individual factors to an emphasis on environmental factors, and rigorous evaluations of outcomes-driven interventions.

## Supplementary Information



## Figures and Tables

**Table 1:** Primary studies of wellness interventions for residents in emergency medicine.

Primary Author & Year	Title	Population & Design	Type of Intervention	Description of Intervention	Measured Outcome(s)	Kirkpatrick Levels	Main Findings
Calder-Sprackman (2018)	Ice Cream Rounds: The adaptation and evaluation of a peer-support wellness rounds in an emergency medicine resident training program	Case report at single program	Peer support	Voluntary attendance at confidential, peer support sessions (rounds) Led by two peers who received training at Faculty of Medicine’s Wellness Program Discussions topics: difficult patient encounters, poor patient outcomes, residency challenges, ethical issues	Overall attendance Feedback from pilot sessions	Level 1	Residents reported rounds were a helpful to discuss important issues with colleagues Peer-support wellness rounds are effective for debriefing and can be easily adopted
Castillo (2018)	Trainees and Faculty Healing Together: A Resident- and Faculty-directed Wellness Initiative for Emergency Medicine Residents	Case study at dual-campus program	Curriculum	All-day wellness initiative “theme day” 2 lectures by EM faculty on existing research and adverse outcomes associated with burnout 5 stations on clinician wellness led by peer resident facilitator and faculty (narrative medicine, burnout assessment, needs analysis, communication strategies) Group social activity Open forum for reflection	Rating of applicability of each station Overall feedback on teaching structure of leaders	Level 1	87% enjoyed structure 75% felt theme day was valuable 74% felt all 5 stations applicable and useful Open responses for combating burnout (stacked shifts, cross-dept communication, faculty menotrship)
Comp (2018)	ED Stories: Online Emergency Medicine Residency Community Building and Wellness Initiative	Case report at single program	Storytelling	Utilized Slack platform to engage residents in a virutual discussin covering difficult situations, patient interactions, tips on how to succeed in challenging circumstances Goal to create “safe space” free of judgement and criticism and to erve as an outlet	Number of participants, interactions, unique written posts, comments, and other reactions Measured after month 1 and in subsequent 5 months	Not applicable	1st month - 54 residents and staff invited, 17 participants (11 residents, 5 attendings, 1 coordinator); 81 total interactions, 10 unique posts, 17 comments, 54 other reactions Next 5 months - 54 invited, 24 participants (15 residents, 8 attendings, 2 coordinators); 131 interactions, 14 posts, 22 comments, 95 other reactions
Hart (2019)	Does Implementation of a Corporate Wellness Initiative Improve Burnout?	Pre/Post-Assessments at single program	Employee Assistance Program (EAP)	Corporate wellness program (The Happiness Practice) successful with other healthcare providers (e.g. RNs, hospital leadership) implemented during residency conference hours 6 monthly didactic sessions on core principles (be conscious, honor feelings, release control in favor of empowerment, co-create what works now, learn life lessons) Led by the former business executives that founded THP Optional small group evening social discussions between sessions at resaurants 1^st^ didactic session was 1 hour, subsequent were 15 min sessions (revised per resident feedback on 1^st^)	MBI (EE, PA, DP) before and after Reactions after each session Overall satisfaction with program Subjective report of burnout	Level 1 Level 4	Trend toward increased overall burnout scores in EE and DP, improved PA Overall same trends seen in PGY 1, 2, and 3+ years Low overall satisfaction (1.5/5) 17/99 written responses coded as negative (instructors had poor understanding of residency stressors and EM work, not relevant, not tailored toward healthcare professionals, would rather spend conference hours learning about topics related to medicine)
Lefebvre (2018)	Resident Physician Wellness Curriculum: A study of efficacy and satisfaction	Pre/Post-surveys at single program	Curriculum	Multi-faceted wellness curriculum Faculty-derived (F-RWC) and parallel resident-derived (R-RWC) curriculum added after 1^st^ year	BRWP SF-8 Health Survey, satisfaction scale Measured at baseline, after 1 year F-RWC, after 1 year combined F-RWC and R-RWC	Level 1 Level 4	Significant improvement in wellness with addition of parallel R-RWC to F-RWC Positive satisfaction scores of R-RWC Mental wellness initiatives were best-attended events (monthly music playlists, wellness seminar at annual retreat, Christmas gift donation drive, blood donation drive, Remembrance Day perspective material)
Moriarty (2017)	Improving resident wellness using a moderated faculty panel	Post-intervention survey at single program	Panel discussion	4 EM faculty participated in 30 min panel during resident conference, answered wellness-focused questions submitted by residents	Satisfaction survey	Level 1	23/27 completed survey Majority felt panel was helpful for advice on work-life balance and coping with stressful life events; indicated they wanted more in the future
Poonja (2018)	Sleep and Exercise in Emergency Medicine: An observational pilot study exploring the utility of wearable activity monitors for monitoring wellness	Pre/Post- survey at single EM program with 2 training sites	Wearable fitness trackers	Residents wore wearable fitness tracker (FitBit) for one 4-week rotation Collected data on sleep quality (number of awakenings), sleep quantity (number of minutes asleep), exercise quality (number of minutes of vigorous activity), exercise quantity (number of daily steps)	PWS FitBit recorded data	Level 1 Level 3 Level 4	No statistically significant correlation between resident PWS scores, sleep interruptions, average daily sleep minutes, daily step count, or average daily active minutes for the sample overall 1^st^ year residents and residents from years 2–5 reported different median PWS scores of 13.9 and 17.1, respectively Step counts may correlate with wellness in participants in year 1, quantity of sleep may have an association with wellness in participants in years 2–5 Concluded that wearable tracking technology wasn’t an effective intervention
Willaimson (2019)	The Implementation of a National Multifaceted Emergency Medicine Resident Wellness Curriculum is Not Associated with Changes in Burnout	Pre/Post-evaluation, RCT at 10 EM programs	Curriculum	Year-long multifaceted wellness curriculum implemented at half of sites (5 of 10)	MBI administered at baseline, 6 months, 1 year	Level 4	MBI scores were stable over time, implementation of curriculum was not associated with global changes in burnout When measured as continuous variable, control sites scored lower on depersonalization at baseline and final survey, higher mean personal achievement scores at second survey When measured as dichotomous variable, no differences in global burnout at any time and no change in burnout scores from baseline or over time for control or intervention sites

*ED*, emergency department; *RN*, registered nurse; *MBI*, Maslach Burnout Inventory; *EE*, emotional exhaustion; *PA*, personal accomplishment; *DP*, depersonalization; *PGY*, post-graduate year; *BRWP*, Brief Resident Wellness Profile; *EM*, emergency medicine; *PWS*, Perceived Wellness Survey.

**Table 2:** Assessment tools used to evaluate intervention effectiveness.

Assessment tool	Description
Brief Resident Wellness Profile^23^	Mood faces graphical rating item and 6-question subscale designed to measure resident’s sense of professional accomplishment and mood
Perceived Wellness Scale^24^	36-item survey measuring an individual’s perception of their own wellness
Short Form Health Survey (SF)^25^	8-item abbreviated version (SF-8) of a generic 36-item, health-related quality of life survey instrument
Maslach Burnout Inventory^26^	22-item survey measuring burnout along three subscales: emotional exhaustion, depersonalization, lack of personal achievement
