# Supplementary material for: Wellness Interventions in Emergency Medicine Residency Programs: Review of the Literature Since 2017
Source: West J Emerg Med. 2020 Dec 19;22(1):7–14. doi: 10.5811/westjem.2020.11.48884 (PMC7806318; doi:10.5811/westjem.2020.11.48884)
Supplement: Supplementary file 1 [file wjem-22-7-s001.docx]

**Appendix 1: Literature Reviews and Best Practice Summaries**

| Primary Author & Year | Title | Key Findings |
| --- | --- | --- |
| Arnold (2017) | An Evidence-based, Longitudinal Curriculum for Resident  Physician Wellness: The 2017 Resident Wellness Consensus Summit | Describes development of wellness curriculum as part of ALiEM Wellness Think Tank with goal of creating a guiding framework for EM residency programs and potentially other specialties to improve wellness and promote a culture of wellness  First phase via Slack forum by Wellness Curriculum Development working group, performed literature review, identified themes then incorporated into 10 core topics, group then developed a module, recommended approach, and list of additional resources/readings for each topic  Second phase in person at Resident Wellness Consensus Summit, presented preliminary curriculum, received feedback and added 7 topics that contribute to personal wellness (especially in EM); total of 17 topics - Introduction, Why Wellness Matters, Self-Care Series (7), Physician Suicide, "I Need Help," Clinical Care Series (4), Wellness in the Workplace, Dealing with Medical Errors and Shame |
| Ross (2017) | Strategies to Enhance Wellness in Emergency Medicine Residency Training Programs | Strategies for residents:  Sleep, exercise, and nutrition: maintain “anchor sleep,” eliminate true overnight shift in favor of “casino shifts” (6P-3A, 3A-12P), clockwise shift rotation to promote circadian stabilization, goal of at least 150 minutes of exercise per week of moderate-intensity aerobic activity; balanced meals and healthy snacking, department/program support and provision of healthy food options  Personal health: establish care with primary care provider, annual physical exams, encouragement from program/system to seek help and support for mental and physical well-being  Life outside residency: establish boundaries to maintain work-life balance, set aside 1 hour/day to decompress and refocus, prioritize time for socialization and family, scale back on obligations, finish charting on shift, complete email and administrative duties while at hospital, plan vacations to be work-free  Mindfulness: take time for personal reflection, join a support group, journal, learn meditation  Positivity: support from residency leaders to reflect on negative experiences in positive way, create culture of positivity, recall positive impact made on patients’ lives  Strategies for EM residency programs:  Training community: make creating and fostering wellness a priority, construct curriculum to optimize resident wellness  Wellness committee and curriculum: program should have committee with select resident and faculty members of leadership team; measure, analyze, disclose, address issues identified by residents; build wellness into budgeted didactic time  Mentorship: formal faculty mentorship program with training of mentors on effective delivery of professional and psychosocial support, peer mentorship through “residency families” to provide informal structure and support  Meaningful service: reduce administrative requirements, provide strategies to manage administrative demands and explain utility for future employment, minimize impact of EMR (e.g. dictation services, scribes, smart texts), publicly recognize resident success (e.g. informal/formal awards, “resident of the month," "great save”)  Feedback: establish culture of respect and positivity across specialties; train educators on provision of constructive, respectful, effective feedback; train residents on how to receive feedback; provide transparent and comprehensive evaluations  Debriefing: provide debriefing with entire team immediately after difficult case, normalize difficult emotions, offer assistance with informal or formal counseling, schedule monthly debriefings  Awareness: train residents to be watchful of subtle changes in peers’ behavior, train residents to self-identify, reduce barriers to counseling (e.g. cost, time, stigma), establish protocol for leadership team follow up with at-risk residents, normalize discussion of stress/burnout/mental health, incorporate wellness and burnout screening surveys  Tribalism: encourage and highlight positive interactions with staff/other services, organize interdisciplinary events, focus on shared values, recognize that all groups are working toward common goal of taking care of mutual patient |
| Ting (2018) | From abstraction to action: Making wellness practical during residency training | Editorial answers 3 questions on practicality of implementing resident wellness intervention  1). How can we integrate curriculum into already full resident schedules? Use residency already protected time at annual retreat, create mentorship program, encourage department to provide healthy snack  2) How can we foster vulnerability within programs? Implement small groups, storytelling, find a "failure friend," become mindful of self-talk and replace negative language with positive  3) How can we learn to say no when saying yes has gotten us to where we are? Ask ourselves if opportunity will bring achievement (external validation) and fulfillment (internal satisfaction), aim to spend 10-20% of time pursuing activities that provide fulfillment to avoid high levels of burnout, use practical language (e.g. "and" instead of "but" statements) |
| Williamson (2017) | Development of an Emergency Medicine Wellness Curriculum | Multi-institution collaboration to meet Accreditation Council for Graduate Medical Education (ACGME) Common Program Requirement  Conducted a needs analysis of EM residents with aim of creating a new wellness curriculum to promote health and resilience, develop lifelong approach to self-care and sustained joy and satisfaction in EM, found that residents did not feel comfortable with their level of knowledge of wellness principles  Developed 12-month curriculum (“Choose Wellness EM”) by integrating previously published and accepted, non-EM curricula and online academic wellness programs  Integrated program at 5 EM residencies  Curriculum covers Hettler’s Six Dimensions of Wellness (Social, Spiritual, Occupational, Physical, Emotional, Intellectual), includes bi-monthly didactics delivered by faculty at resident conference, individual interactive instruction assignments and non-didactic components to improve personal/professional wellness, additional internet-based resources (e.g. websites, apps) |
| Zaver (2018) | Identifying Gaps and Launching Resident Wellness Initiatives: The 2017 Resident Wellness Consensus Summit (RWCS) | Academic Life in Emergency Medicine (ALiEM) Wellness Think Tank (WTT) members involved in Programmatic Initiatives workgroup collected information from EM residency programs with goal of creating a wellness initiative repository.  Identified 36 unique residency wellness initiatives from ALiEM WTT and Chief Resident Incubator communities.  Created a centralized, searchable, online database open to the public and contributor form for future submissions.  Used on general workplace needs-assessment tools on well-being and Kern’s model for curriculum development to create two tools:  Resident-Based Needs Assessment Survey on residency wellness programming to evaluate current initiatives, existing wellness interests, perception of culture of wellness, leadership support for wellness activities  Worksheet on Implementing New Wellness Initiatives to help programs implement new initiatives; part 1 assesses existing resources and previous experiences, part 2 focuses on building a new initiative or strategy |
